# Effectiveness of Integrated Neurocognitive Therapy on Cognitive Impairment and Functional Outcome for Schizophrenia Outpatients

**DOI:** 10.1155/2018/2360697

**Published:** 2018-10-21

**Authors:** Andreana De Mare, Miriam Cantarella, Giovanni Galeoto

**Affiliations:** ^1^Sapienza University of Rome, Italy; ^2^Department of Public Health and Infection Disease, Sapienza University of Rome, Italy

## Abstract

Cognitive impairment is highly prevalent in patients with schizophrenia and schizoaffective disorder. Many interventions have been developed to treat cognitive deficit, since it has a strong impact on functional outcome; however, there are no integrated interventions targeting multiple neuro- and social-cognitive domains with a particular focus on the generalization of the effects of therapy on the functional outcome. Recently, a group of experts has developed a cognitive remediation group therapy approach called Integrated Neurocognitive Therapy (INT), which includes exercises to improve the MATRICS (Measurement and Treatment Research to Improve Cognition in Schizophrenia) neuro- and social-cognitive domains. This systematic review and meta-analysis aimed to assess the efficacy of this approach. We conducted a search of PubMed, Scopus, Web of Science, and PsycINFO to select primary studies evaluating INT in schizophrenic and schizoaffective patients. The primary outcomes of the meta-analysis included negative and positive symptoms and global functioning. Two randomized controlled trials met inclusion criteria. A total of 217 participants were included. Based on the results from the Positive and Negative Syndrome Scale (PANSS), a significant pooled effect size was observed for negative symptoms, which demonstrated not only an improvement in the patients treated immediately after therapy but also a permanence of positive results at a 9–12-month follow-up. On the other hand, no significant effect size was observed for positive symptoms. In addition, a significant pooled effect size was found for Global Assessment of Functioning (GAF), which shows how INT's integrated approach has lasting positive implications on patients' functional outcome. We concluded that INT might be an effective treatment for negative symptoms and global functioning in patients with schizophrenia, compared to treatment as usual (TAU).

## 1. Introduction

Cognitive deficits are one of the most important features in schizophrenia with significant consequences on patients' psychosocial functioning [1]. According to some studies, about 98% of schizophrenic patients have an impairment in a wide range of cognitive functions [2], including working memory, attention, processing speed, visual and verbal learning with substantial deficit in reasoning, planning, abstract thinking, and problem solving [3].

Two recent meta-analyses demonstrate that a minor cognitive impairment is present at the onset of the disease. The authors of these studies reported the values of premorbid IQ (Intelligence Quotient) in subjects who later developed schizophrenia, showing that years before the onset of psychotic symptoms, individuals with schizophrenia, as a group, demonstrated mean IQ scores approximately one-half of a standard deviation below that of healthy comparison subjects. [4, 5].

In support of the observation that social and occupational impairment in schizophrenic patients is strongly correlated with neurocognitive deficits, there has been an increasing interest in the development of new pharmacological agents to improve cognition, and consequently an interest in determining which cognitive domains should be represented in a battery of standardized neuropsychological tests, in order to evaluate the efficacy of pharmacological treatments. From these considerations, the MATRICS project (Measurement and Treatment Research to Improve Cognition in Schizophrenia) starts [6].

In order to develop a standardized cognitive battery to foster the evaluation of cognitive enhancement interventions, the group of experts of the MATRICS project identified the major separable cognitive impairments in schizophrenia. Summing up the data of a large number of studies, these authors have identified the cognitive areas that are altered in patients by dividing them into seven separate cognitive domains that were replicable across studies: speed of processing, attention/vigilance, working memory, verbal learning and memory, visual learning and memory, reasoning and problem solving, and social cognition [7].

However, to date, it has been seen that antipsychotic drugs have an effect on the main symptoms of the disease but not on these cognitive domains, which explains the increasingly widespread use of cognitive remediation for schizophrenia [8]. Cognitive remediation techniques are defined as interventions based on behavioural training that aims to improve cognitive processes in a long-lasting and generalizable way [9]. These interventions can be classified according to two models: compensatory and reparative. Compensatory interventions try to bypass the deficit and compensate for it by relying on intact cognitive skills and environmental resources, promoting an adaptation of the context in which the patient lives and an adaptation of his behaviour to the specific situation, while restorative interventions try to correct and improve the deficit by drill and practice exercises. The restorative techniques are based on neuroscientific knowledge according to which the neuronal processes compromised can be repaired through repeated exercise, which leads to a restoration of those neuroanatomical connections linked to neuropsychological abilities. This particularly concerns the white matter pathways which, as known through many studies about imaging and MR techniques in schizophrenia, play an important role in the neuropathology of schizophrenia, and are likely related to clinical symptoms observed in this disorder [10]. We must also remember that the restorative model may utilize either a bottom-up (cognitive recovery of elementary skills to get to the most complex ones) or a top-down (use of complex skills to improve indirectly the simplest ones) approach [11].

Over the last few years, different structured protocols of cognitive remediation have been proposed and elaborated, which can be distinguished by the mode of application (individual or group; computerized or paper and pen; constant presence of the therapist) or by whether they are primarily based on repeated execution of specific tasks, or on the development and learning of new strategies [9].

Some recent meta-analyses have shown that cognitive remediation interventions have positive effects not only on cognitive performance, but also on psychosocial functioning and, to a small degree, on symptoms that tend to disappear at the first follow-up [12, 13].

Studies reported that social cognition (comprising emotional perception and social knowledge), is related to both neurocognition and functional outcome [14]. Although it would suggest that an integrated treatment of neuro- and social cognition may produce better generalization effects on functional outcome than neuro- or social-cognitive therapy alone, only a few interventions that combine the rehabilitation of both cognitions have been developed.

Furthermore, as there is no evidence that boosting one cognitive domain might improve functional outcome more than another [15], an approach targeting multiple cognitive domains may be of benefit for most schizophrenia patients. Nevertheless, none of the contemporary approaches integrates all cognitive domains identified by the MATRICS project experts. [16].

Only recently did a group of experts from the University of Bern develop such an approach—called Integrated Neurocognitive Therapy (INT)—which combines both neurocognition and social cognition by developing specific interventions for each MATRICS domain. In this way, based on the results of current meta-analyses [12, 13, 17], a significant improvement in cognitive domains, symptoms, and functional outcome is assumed [16, 18].

This recent intervention is an evolution of the previous cognitive remedy intervention called Integrated Psychological Therapy (IPT) that aimed to improve specific cognitive functions and the acquisition of social skills through five subprograms: cognitive differentiation, social perception, verbal communication, social skills, and interpersonal problem-solving [19].

The INT is composed of four modules, each of which focuses on different cognitive domains and on social cognition: Module A takes into account the processing speed, attention and perception of emotions; Module B concerns verbal and visual learning and memory, social perception and theory of mind; Module C is about reasoning, problem solving, and “social schemes”; and Module D trains working memory and the ability to attribute appropriate meanings. Some exercises within the four modules use a computer, particularly the Cogpack computer program [20].

From the preliminary research, we found two articles [16, 18] that tested the effectiveness of INT based on randomized control trials. Then, we did a systematic literature review and conducted a meta-analysis to quantify the improvement of patients following therapy and the permanence of positive effects at follow-up after about one year.

## 2. Material and Methods

### 2.1. Search Strategy

We searched four electronic databases—PubMed, Web of Science, Scopus and PsycINFO— from inception to May 2018. The search terms that we used were “Integrated Neurocognitive Therapy”, “Schizophrenia”, and “Randomized controlled trial”. No language restrictions were imposed and we applied the following inclusion criteria: (1) randomized controlled trial that (2) evaluated INT versus TAU (treatment as usual) in (3) adult patients with diagnosis of schizophrenia or schizoaffective disorder.

### 2.2. Studies Selection and Quality Assessment

Two authors independently researched the articles using the search terms and independently screened titles and abstracts according to the eligibility criteria to select relevant studies. The quality of the included studies was assessed by using Cochrane Collaboration's tool for assessing risk of bias by RevMan [21] and by extracting PEDro (Physiotherapy Evidence Database) scores from the PEDro website. Each score on the PEDro website is generated by two accredited raters scoring the trial; any discrepancies in rating are resolved by a third accredited rater (https://www.pedro.org.au/).

### 2.3. Statistical Analysis

We performed a meta-analysis using Review Manager software (RevMan, the Cochrane Collaboration).The mean difference (MD) was used as the effect size for continuous outcomes. A fixed-effect model was used, as we expected a fixed effect-size from the studies. The overall effect sizes were calculated based on the pooled proportions and 95% confidence intervals (CIs). The differences between the studies were calculated through the overall effect size (Z), with a statistical significance threshold of p <0.05. The data used for statistical analysis were divided according to two points in time: first, we considered the posttreatment results after 15 weeks and, then, the results obtained at a follow-up that included a range of 9-12 months.

### 2.4. Outcome Measures

The primary outcomes of interest included negative and positive symptoms measured by PANSS (positive and negative syndrome scales) and global functioning assessed by GAF (Global Assessment of Functioning Scale). We collected both the posttreatment outcome at 15 weeks and the follow-up outcome creating a range from 9 to 12 months.

## 3. Results

### 3.1. Search Results

**Figure 1:**
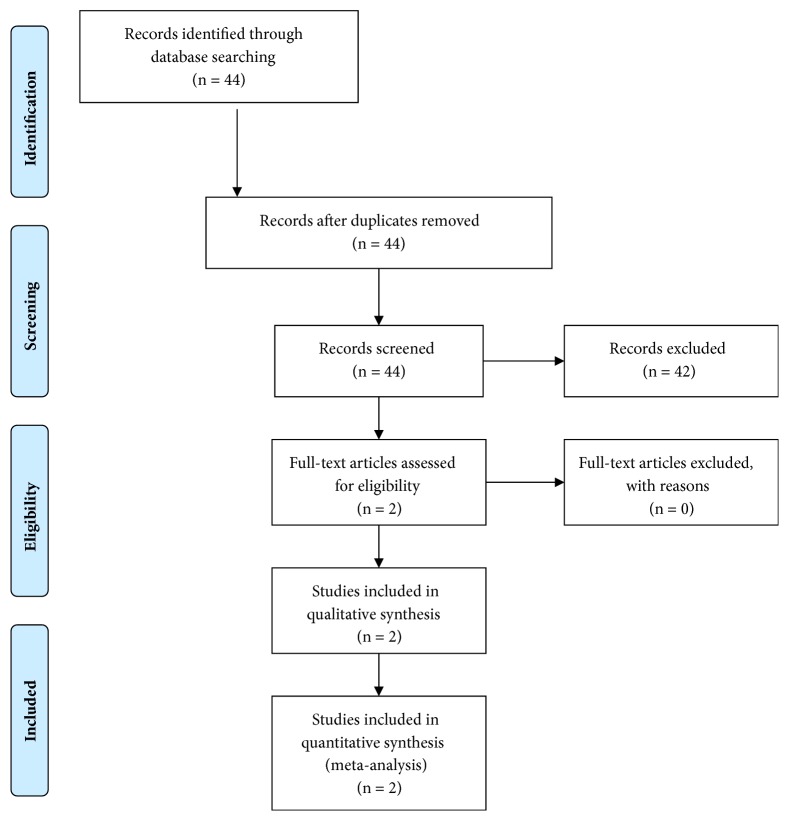
Flow-chart.

The study selection process is diagrammed in Figure 1. A total of 44 records were identified and screened through the initial search strategy, and a total of 42 records were excluded based on irrelevant titles and abstracts. Two RCTs (randomized controlled trials) met the eligibility criteria.

### 3.2. Characteristics of Included Studies

A summary of studies characteristics is shown in Table 1. The two studies were published from 2015–2017 and included 217 participants: 155 of them were male and 62 were female. The mean age across all participants was 34.9 years (age range: 18-50). The intelligence quotient (IQ) mean, assessed by the Reduced Wechsler Intelligence Test (WIP), was 102.7; the mean duration of illness was 10.4, while the number of hospitalizations was 4.2. The chlorpromazine equivalent doses mean was 422.5.

The studies were from Switzerland, and in both of them, the modalities of intervention were the same: 30 biweekly sessions of INT, with each session lasting 90 minutes. Likewise, each RCT compared the intervention groups to the TAU groups; TAU is defined as standard care which includes a broad array of intervention used in clinical practice for schizophrenic patients.

#### 3.2.1. Trial Quality

**Figure 2:**
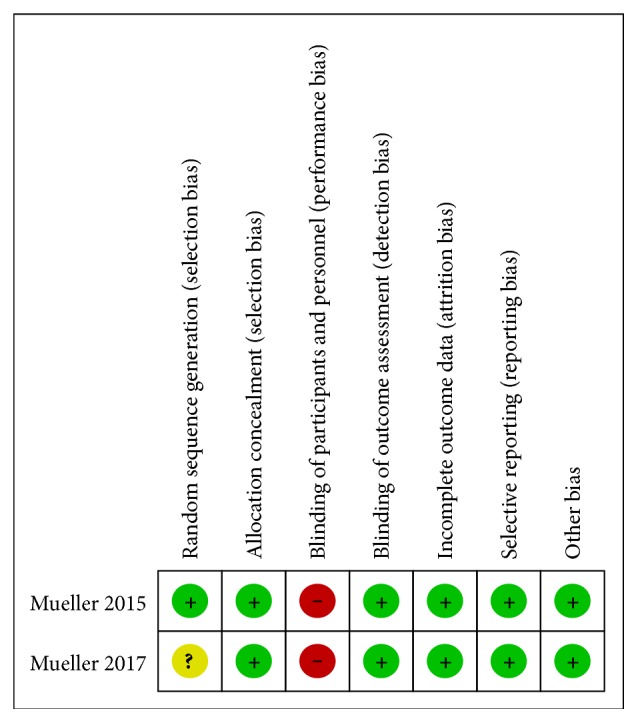
Quality appraisal. + (green), low risk of bias; ? (yellow), unclear risk of bias; − (red), high risk of bias.

The Cochrane collaboration's tool for assessing risk of bias was used to assess risk of bias of each study (Figure 2). Both studies were assigned high risk in blinding of participants and personnel (performance bias) due to the nature of treatment; on the other hand, risk of all other bias was low, apart from selection bias risk, which was unclear in Mueller 2017 trial because of a missing adequate description of randomisation.

We also used the PEDro scores to assess the quality of included studies (Table 2). According to the PEDro criteria, the quality of the studies can be classified as three ranges: low quality (scores 0-3), medium quality (scores 4-7), and high quality (scores 8-10). The score of 10 reflects the best quality.

The score of our papers was 8, due to missing blind participants and therapists. In both Mueller's studies, the dropout rate was <15% at each point in time: for the 15-week therapy phase and 10.2% dropped out in the 2015 study, while 9.8% dropped out for the 2017 one.

For the 9-12-month follow-up phase, 13.6% dropped out in the 2015 study, while 12.7% dropped out for the 2017 one. Both RCTs were rated as high-quality studies. The quality assessments were initially completed by a single reviewer and then checked for accuracy by one other reviewer.

### 3.3. Meta-Analysis of Primary Outcomes

Data from the PANSS Negative and Positive Syndrome Scale and the Global Assessment of Functioning Scale were included in the meta-analysis.

### 3.4. Effectiveness of INT on Negative Symptoms Assessed by PANSS

**Figure 3:**
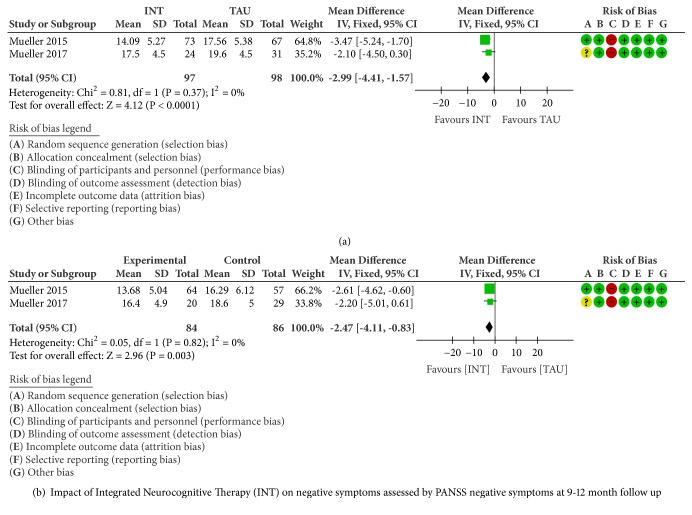


Based on the results from PANSS negative symptoms, a significant pooled effect size was observed after 15 weeks (MD -2.99, 95%CI -4.41 to -1.57, and P<0.0001; Figure 3(a)) and at 9-12-month follow-up (MD -2.47, 95%CI -4.11 to -0.83, and P=0.003; Figure 3(b)), with no evidence of statistical heterogeneity (I2=0%).

Since in this scale, lower scores indicate more improvements, INT produced a reduction in negative symptoms, which was maintained at the follow up, as shown in Figures 3(a) and 3(b).

### 3.5. Effectiveness of INT on Positive Symptoms Assessed by PANSS

**Figure 4:**
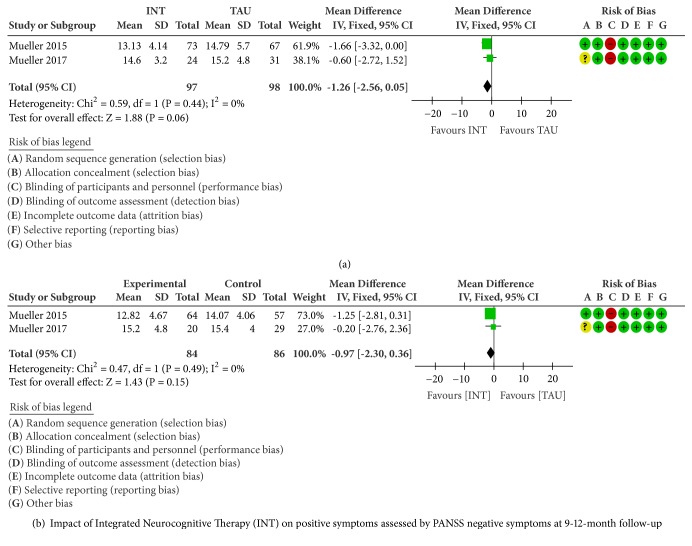


**Figure 5:**
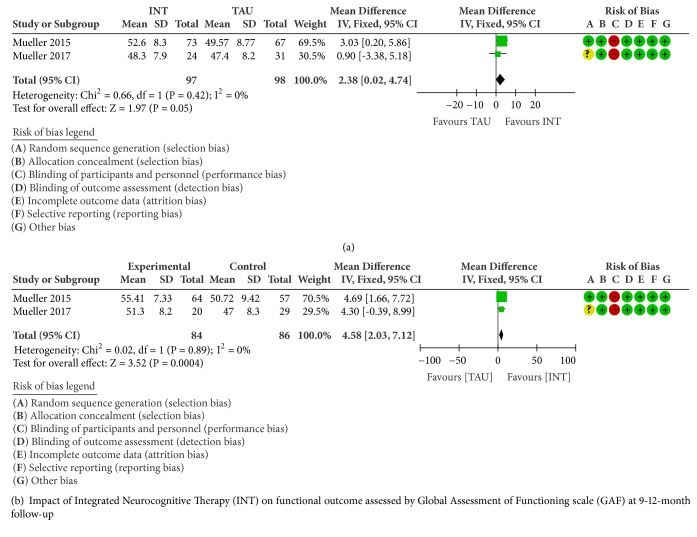


No significant pooled effect sizes were found for PANSS Positive symptoms after 15 weeks (MD -1.26, 95%CI -2.56 to 0.05, and P=0.06; Figure 4(a)), as well as 9-12 month-follow-up (MD -0.97, 95%CI -2.30 to 0.36, and P=0.15; Figure 5(a)) with no evidence of statistical heterogeneity (I2=0%).

### 3.6. Effectiveness of INT on Functional Outcome Assessed by Global Assessment of Functioning Scale (GAF)

A significant pooled effect size was observed for GAF after 15 weeks (MD 2.38, 95%CI 0.02 to 4.74, and P=0.05; Figure 5(a)), and at 9-12-month follow-up (MD 4.58, 95%CI 2.03 to 7.12, and P=0.0004; Figure 5(b)) with no evidence of statistical heterogeneity (I2=0%).

## 4. Discussion

Although there are many studies on various cognitive remediation approaches, there are only two trials conducted specifically on INT which tested its efficacy. Both the RCTs we found demonstrated the improvement of the patients in different deficit areas, so we summarized the data to integrate the results; we hoped to obtain a single quantitative estimate index that would allow us to draw stronger conclusions than those drawn on the basis of each single study.

It would be important to have access to a greater amount of data to conduct a meta-analysis and, therefore, a more exhaustive synthesis. This would allow the creation of stronger evidence than that deriving from the clinical studies, which is important considering the impact this type of intervention could have on the functional recovery of schizophrenic patients.

In this systematic review and meta-analysis, we identified two eligible studies assessing the effect of INT on schizophrenic or schizoaffective patients. From the meta-analysis, we found that INT had a significant impact upon negative symptoms and functional outcome, compared to TAU, as demonstrated by improved scores in both assessment scale used. However, no significant improvement was observed in positive symptoms.

INT is a new cognitive remediation group approach. It incorporates all 11 neuro- and social cognitive domains defined by National Institute of Mental Health-Measurement and Treatment Research to Improve Cognitions in Schizophrenia (NIMH-MATRICS) into four therapy modules. Each module starts with interventions on neurocognitive domains and is followed by interventions on social cognition [18].

The meta-analysis provides evidence that INT may be effective in treating schizophrenic patients' cognitive impairment. It could decrease negative symptoms, while pharmacological treatments have shown limited success for alleviating them. On the other hand, the most important finding of our study was that INT produced robust and durable generalization effects on functional outcome, as assessed by the GAF, which even increased over time [18].

### 4.1. Study Limits

This study has several limitations. First, due to the limited number of existing RCTs on INT, this meta-analysis was based only on data extracted from the two RCTs. Second, even if other outcomes assessed in the two RCTs could be comparable, in one article, there were some scales including standardized scores, so the comparison could not be realized.

Due to the limited number of included studies, these results should be confirmed by further large-scale RCTs which focus on INT's effects on schizophrenic patients.

### 4.2. Conclusions

This study was conducted by a research group composed of medical doctors and rehabilitation professionals from the Sapienza University of Rome and from the Rehabilitation & Outcome Measure Assessment (ROMA) association. In the last few years, the ROMA association has dealt with the validation of many outcome measures in Italy. [22–37]

In summary, despite the limitations, our results suggest that INT is a feasible and effective approach; it has the potential to improve neuro- and social cognition and, in a lasting way, to influence positively functional outcome in schizophrenia outpatients. However, the durability of the effects on the reduction of positive symptoms was not found. Given the current scarcity of RTCs, further studies are needed to compare between different patient populations and to produce stronger evidence in this specific rehabilitative setting.

## Figures and Tables

**Table 1:** Characteristics of the studies.

*Mean*	Mueller 2015 N=156	Mueller 2017 N=61
Age	34.2	35.6
Gender (N male)	108	47
IQ mean	103.9	101.6
Duration of Illness (years)	10	10.8
Number of hospitalizations	4.1	4.3
Chlorpromazine equivalent doses	439.1	406

**Table 2:** Pedro scores for included papers (n=2) extracted from website www.pedro.org.au.

	Mueller 2015	Mueller 2017
Eligibility Criteria	YES	YES
Random allocation	YES	YES
Concealed allocation	YES	YES
Groups similar at baseline	YES	YES
Participant blinding	NO	NO
Therapist blinding	NO	NO
Assessor Blinding	YES	YES
<15% dropouts	YES	YES
Intention to treat analysis	YES	YES
Between-group difference reported	YES	YES
Point estimate and variability reported	YES	YES
Total (0-10)	8	8
